# Military veterans and civilians’ mental health diagnoses: an analysis of secondary mental health services

**DOI:** 10.1007/s00127-022-02411-x

**Published:** 2022-12-22

**Authors:** Charlotte Williamson, Laura Palmer, Daniel Leightley, David Pernet, David Chandran, Ray Leal, Dominic Murphy, Nicola T. Fear, Sharon A. M. Stevelink

**Affiliations:** 1 King’s Centre for Military Health Research, King’s College London, Weston Education Centre, London, SE5 9RJ UK; 2 Department of Psychological Medicine, Institute of Psychiatry, Psychology and Neuroscience, King’s College London, London, SE5 8AF UK; 3 Research Department, Combat Stress, Leatherhead, KT22 0BX UK; 4 Academic Department of Military Mental Health, King’s College London, Weston Education Centre, London, SE5 9RJ UK

**Keywords:** Mental health, Military, Armed forces, Veteran, Civilian, Secondary healthcare

## Abstract

**Purpose:**

Healthcare provision in the United Kingdom (UK) falls primarily to the National Health Service (NHS) which is free at the point of access. In the UK, there is currently no national marker to identify military veterans in electronic health records, nor a requirement to record it. This study aimed to compare the sociodemographic characteristics and recorded mental health diagnoses of a sample of veterans and civilians accessing secondary mental health services.

**Methods:**

The Military Service Identification Tool, a machine learning computer tool, was employed to identify veterans and civilians from electronic health records. This study compared the sociodemographic characteristics and recorded mental health diagnoses of veterans and civilians accessing secondary mental health care from South London and Maudsley NHS Foundation Trust, UK. Data from 2,576 patients were analysed; 1288 civilians and 1288 veterans matched on age and gender.

**Results:**

Depressive disorder was the most prevalent across both groups in the sample (26.2% veterans, 15.5% civilians). The present sample of veterans accessing support for mental health conditions were significantly more likely to have diagnoses of anxiety, depressive, psychosis, personality, and stress disorders (AORs ranging 1.41–2.84) but less likely to have a drug disorder (AOR = 0.51) than age- and gender-matched civilians.

**Conclusion:**

Veterans accessing secondary mental health services in South London had higher risks for many mental health problems than civilians accessing the same services. Findings suggest that military career history is a key consideration for probable prognosis and treatment, but this needs corroborating in other geographical areas including national population-based studies in the UK.

**Supplementary Information:**

The online version contains supplementary material available at 10.1007/s00127-022-02411-x.

## Introduction

The most up-to-date estimate of the United Kingdom’s (UK) military veteran population is approximately 2.5 million, equivalent to around 5% of household residents aged 16 years and above [1]. A veteran in the UK is defined as an individual who has served a minimum of one day in the UK Armed Forces but no longer serves [2]. The National Health Service (NHS) in the UK is responsible for the provision of physical and mental healthcare to veterans, and this care is recorded in local, regional and national Electronic Healthcare Records (EHRs) [3]. EHRs are structured (i.e. clinical diagnosis) and unstructured (i.e. free text), and can be used to evaluate disease prevalence, for surveillance, to perform epidemiological analyses, to investigate quality of care, and to improve clinical decision making [4, 5]. Currently, there is no national marker in UK EHRs to identify veterans, nor is there a requirement for healthcare professionals to record it. This makes it difficult to evaluate the unique healthcare needs of those who have served in the military, including those related to mental health which have received much attention in recent years by the media, politicians and the general public [6].

Previous research estimates that approximately 6–22% of UK veterans experience a mental disorder, including post-traumatic stress disorder (PTSD; 6.2%), common mental disorders (CMDs; 21.9%) like anxiety and depression, and alcohol misuse (10%) [7]. The demands of military service impact on personnel both during (serving) and after service (veterans) and can contribute to a deterioration of physical and/or mental health [7]. Specific occupational stressors, such as deployment history and combat experience, play a role in the risk of mental disorders and alcohol use disorder amongst veterans [7]. These stressors are compounded by other factors including childhood adversities, which appear to be higher in UK veteran populations compared to the general (civilian) population [8, 9].

In the UK, there have been a limited number of direct comparisons between veteran and civilian populations. There is evidence to suggest that the prevalence of mental disorders and alcohol misuse are higher amongst veterans compared to civilians using self-report mental health measures [10]. Rhead et al. found that UK veterans who served at the time of recent military operations were more likely to report a significantly higher prevalence of health issues compared to civilians, for instance, PTSD (8% vs. 5%), CMDs (23% vs. 16%) and alcohol misuse (11% vs. 6%) [10].

In addition, another UK study explored the patterns of alcohol misuse in treatment-seeking veterans measured using the Alcohol Use Disorder Identification Test (AUDIT; [11]) [12]. Murphy and Turgoose found that treatment-seeking veterans had higher levels of alcohol misuse and reported more alcohol dependence and alcohol-related harm than the general population [12]. These UK treatment-seeking veterans reported higher levels of hazardous (AUDIT score of 8 +) or harmful (AUDIT score of 16 +) alcohol misuse compared to the general population (42% vs. 38% and 22% vs. 6%, respectively), and reported more than double the percentage of alcohol-related harm compared to the general population (37% vs. 15%, respectively) [12].

A substantial number of UK veterans (up to 69%) do not appear to seek formal medical help and support when needed [13]. In the UK, there is a lack of empirical research focussing upon the mental health characteristics of veterans accessing secondary mental health care when compared to civilian counterparts. The aim of this study was to compare the sociodemographic characteristics and recorded mental health diagnoses of an age- and gender-matched sample of UK military veterans and civilians accessing secondary mental health services, using data drawn from EHRs.

## Methods

### Study design and sample

The study compared veterans and civilians accessing mental health treatment from the South London and Maudsley (SLaM) NHS Foundation Trust, a secondary and tertiary mental healthcare provider serving a geographical catchment of approximately 1.3 million UK residents from four London boroughs (Lambeth, Southwark, Lewisham, and Croydon) [14]. As there is no marker to identify veterans in EHRs, this study employed the Military Service Identification Tool (MSIT) [15], a machine learning computer tool, to identify military veterans using probabilistic modelling of free-text clinical notes. The MSIT was found to have high precision and accuracy for correctly detecting veterans in EHRs, with an overall accuracy rating of 97% [15, 16].

The MSIT was executed across 150,000 patients, each with three randomly selected free-text clinical notes extracted from the SLaM NHS Trust. Relevant descriptive variables to address the study aims were extracted from the SLaM NHS Trust system using the Clinical Record Interactive Search (CRIS) system [14, 17], a system that extracts EHRs and de-identifies records for use in research. The sample of probable veterans identified were matched on age and gender to a civilian sample to form the SLaM-Military-Civilian cohort. The research team manually inspected free-text clinical notes of each patient in the cohort to validate civilian or veteran status.

All patients (overall *N* = 2576; civilians *n* = 1288; veterans *n* = 1288) accessed treatment within the SLaM NHS Trust between 2007 and 2018 and were under 64 years of age on 1st January 2007. Age exclusion was implemented to ensure that those who were likely to have served during the era of National Service (those who served prior to 1963) were excluded on the basis that this subgroup had not voluntarily enlisted into the military.

### Data source and measures

The variables extracted from the SLaM NHS Foundation Trust CRIS system included sociodemographic characteristics, military service characteristics, and recorded mental health diagnoses. The extracted sociodemographic characteristics included age (continuous variable); gender (male/female); deceased status (yes/no); ethnicity (Asian or Asian British/African, Caribbean or Black British/White British/any other ethnicity); marital status (married or relationship/single, separated, divorced or widowed); living arrangements (living alone/with parents/with a partner and, or children/with other relatives/other); and deprivation status (least deprived/middle/most deprived).

Employment status, housing status, benefit status and service branch, i.e. whether patients served in the Royal Navy, Army or Royal Air Force, were extracted from the records. Where missing, these variables and other sociodemographic information were ‘backfilled’ meaning each patient’s free-text clinical notes were manually reviewed by the research team to identify and input relevant information. Average completeness of these variables prior to backfilling was 63.0%, this increased to 75.5% after backfilling. Variables that still had a large percentage of missing data (> 10%), such as employment status, housing status, benefit status and service branch, were excluded from the final analyses. However, serving status (UK/ overseas) was well-populated and included in the analyses. International Classification of Diseases version 10 (ICD-10) mental health diagnoses recorded for each patient between 2007 and 2018 were extracted from EHRs; a full break-down can be found in Supplementary Table 1. In the UK, this type of information is structured to enable reimbursement of treatment costs to healthcare providers [3], resulting in full completeness, therefore no backfilling was required. Recorded mental health diagnoses were categorised into alcohol, anxiety, depression, drug, personality, psychosis, and stress disorder, with the additional categories of ‘other’ and ‘not specified’ for those who did not fit into the defined categories.

### Statistical analyses

Descriptive analyses were conducted on the samples’ sociodemographic characteristics, military service characteristics and recorded mental health diagnoses.

Chi-square tests were used to compare the sociodemographic characteristics of veterans and civilians in the sample. Logistic regression analyses were undertaken to investigate the associations between recorded mental health diagnoses (treated as the outcome variable) and civilian or veteran status (treated as the exposure variable), adjusting for ethnicity and marital status. These adjustments were chosen a priori based on existing literature reporting on the sociodemographic determinants of mental health [18]. Upon inspecting the missing data of the exposure variables, only marital status had a high degree of missing data (36.5%). Post hoc sensitivity analyses were carried out for all analyses that used the marital status variable by including the missing data in the model to check that the associations, and their directions, were not impacted. There were no noteworthy differences (data not shown).

Additional logistic regression analyses were conducted to investigate associations between sociodemographic characteristics and recorded mental health diagnoses in the veteran and civilian samples independently. In this analysis, variables with multiple levels (e.g. ethnicity and living arrangements) were collapsed due to low numbers. Results for these analyses are included in Supplementary Tables 2a and 2b. All analyses in this study were conducted using STATA 17.0 (College Station, TX).

### Ethics

Ethical approval for the use of CRIS as an anonymised database for secondary analysis was granted by the Oxford Research Ethics Committee (reference: 18/SC/0372). The current study was approved by the CRIS Patient Data Oversight Committee of the National Institute of Health Research Biomedical Research Centre (reference: 20-049).

## Results

### Study sample characteristics

The total study sample comprised of 2576 patients who were accessing SLaM services; 1288 civilians and 1288 veterans matched on age and gender. Table 1 describes the patient profile and compares the sociodemographic characteristics of the civilian and veteran samples. The mean age of the sample was 40.9 years (SD = 12.7). Overall, the majority of the sample were male (88.4% vs. 11.6% female); a similar composition to the UK Armed Forces (88.8% male, 11.2% female) [19]. In addition, the majority of the sample were still alive (87.6% vs. 12.4% deceased), endorsed white ethnicity (74.9% vs. 25.1% other ethnicities), and were single, separated, divorced or widowed (72.3% vs. 27.7% married or in a relationship). Of the veteran sample, most served in the UK Armed Forces (95.3% vs. 4.7% overseas).

**Table 1:** Characteristics of SLaM-Military-Civilian cohort

	Overall (*N* = 2576)	Civilian (*n* = 1288)	Veteran (*n* = 1288)	*df*	χ^2^ value	*p* value
Age (years), mean (SD)	40.9 (12.7)	40.8 (12.7)	40.9 (12.7)	–	–	–
Gender, *n* (%)						
Male	2276 (88.4)	1138 (88.4)	1138 (88.4)	–	–	–
Female	300 (11.6)	150 (11.6)	150 (11.6)			
Deceased, *n* (%)						
Yes	320 (12.4)	154 (12.0)	166 (12.9)	1	0.51	0.473
No	2256 (87.6)	1134 (88.0)	1122 (87.1)			
Ethnicity, *n* (%)						
Asian/Asian British	79 (3.5)	51 (5.0)	28 (2.3)	3	14.47	0.002*
African/Caribbean/Black British	305 (13.8)	144 (14.0)	161 (13.6)			
White British	1,659 (74.9)	764 (74.5)	895 (75.3)			
Any other ethnicity	172 (7.8)	67 (6.5)	105 (8.8)			
Marital status, *n* (%)						
Married/Relationship	576 (27.7)	227 (24.0)	349 (30.9)	1	12.30	< .001*
Single/Separated/Divorced/Widowed	1503 (72.3)	721 (76.0)	782 (69.1)			
Living arrangements, *n* (%)						
Alone	708 (43.3)	316 (42.9)	392 (43.6)	4	17.26	0.002*
Parents	120 (7.3)	71 (9.6)	49 (5.4)			
Partner and/or children	522 (31.9)	209 (28.4)	313 (34.9)			
Relatives	47 (2.9)	23 (3.1)	24 (2.7)			
Other^1^	238 (14.6)	118 (16.0)	120 (13.4)			
Deprivation status^2^, *n* (%)						
Most deprived (high)	1240 (53.8)	566 (51.8)	674 (55.7)	2	3.52	0.172
Middle	833 (36.2)	411 (37.6)	422 (34.8)			
Least deprived (low)	231 (10.0)	116 (10.6)	115 (9.5)			
Service status, *n* (%)						
Overseas			60 (4.7)			
UK	–	–	1228 (95.3)	–	–	–

Missing data were not included in analyses

^1^This includes staying with foster parents and friends

^2^The index of multiple deprivation is the official measure of relative deprivation for small areas (or neighbourhoods) in England. The index of multiple deprivation ranks every small area in England from 1 (most deprived area) to 10 (least deprived area) based on a range of factors

*p < 0.05

Statistically significant differences were observed between the present sample of civilians and veterans for ethnicity, where veterans were more likely to be of White British ethnicity (75.3% veterans, 74.5% civilians; *p* = 0.002) or other ethnicities (8.8% veterans, 6.5% civilians; *p* = 0.002); marital status, where veterans were more likely to be married or in a relationship (30.9% veterans, 24.0% civilians; *p* < .001); and living arrangements, where veterans were more likely to be living alone (43.6% veterans, 42.9% civilians; *p* = 0.002) or with a partner and/or children (34.9% veterans, 28.4% civilians; *p* = 0.002).

### Recorded mental health diagnoses

Amongst civilians in the present sample, the most prevalent recorded diagnoses were depressive (15.5%), alcohol (13.7%) and drug (12.5%) disorders. A large percentage had disorders characterised as ‘other’ (12.6%) or ‘not specified’ (33.5%) (Table 2). Amongst veterans in the present sample, the most common recorded diagnoses were depressive (26.2%), alcohol (14.1%) and psychosis (13.9%) disorders (Table 2). Again, a large percentage had disorders characterised as ‘other’ (14.8%) or ‘not specified’ (29.2%).

**Table 2:** Recorded mental health diagnoses of the SLaM-Military-Civilian cohort

Mental health diagnosis^1^	*n* (%)	OR (95% CI *p* value)	AOR (95% CI *p* value)^2^
Alcohol disorder	358 (13.9)		
Civilian	176 (13.7)	1	1
Veteran	182 (14.1)	1.04 (0.83–1.30; 0.733)	0.82 (0.65–1.04; 0.101)
Anxiety disorder	186 (7.2)		
Civilian	68 (5.3)	1	1
Veteran	118 (9.2)	1.81 (1.33–2.46; < .001)*	1.41 (1.01–1.96; 0.041)*
Depressive disorder	536 (20.8)		
Civilian	199 (15.5)	1	1
Veteran	337 (26.2)	1.94 (1.59–2.36; < .001)*	1.54 (1.25–1.90; < .001)*
Drug disorder	267 (10.4)		
Civilian	161 (12.5)	1	1
Veteran	106 (8.2)	0.63 (0.48–8.13; < .001)*	0.52 (0.39–0.68; < .001)*
Personality disorder	123 (4.8)		
Civilian	42 (3.3)	1	1
Veteran	81 (6.3)	1.99 (1.36–2.91; < .001)*	1.69 (1.14–2.50; 0.009)*
Psychosis disorder	285 (11.1)		
Civilian	106 (8.2)	1	1
Veteran	179 (13.9)	1.80 (1.40–2.32; < .001)*	1.63 (1.24–2.16; < .001)*
Stress disorder	234 (9.1)		
Civilian	57 (4.4)	1	1
Veteran	177 (13.7)	3.44 (2.53–4.69; < .001)*	2.84 (2.03–3.96; < .001)*
Other	352 (13.7)		
Civilian	162 (12.6)	1	1
Veteran	190 (14.8)	1.20 (0.96–1.51; 0.109)	1.08 (0.84–1.39; 0.535)
Not specified	807 (31.3)		
Civilian	431 (33.5)	1	1
Veteran	376 (29.2)	0.81 (0.69–0.97; 0.020)*	0.96 (0.90–1.03; 0.303)

474 patients had missing diagnoses (18.4%). Some had a diagnosis recorded on their EHR but we were not able to determine its categorisation into ‘Other’ or ‘Not specified’

*OR* odds ratios; *AOR* adjusted odds ratios; *CI* confidence intervals

^1^A full break-down of ICD-10 codes can be found in Supplementary Table 1

^2^Adjusted for ethnicity and marital status

*p < 0.05

Veterans were more likely than matched civilians to have had a recorded diagnosis of stress disorder (AOR 2.84; 95% CI 2.03–3.96), personality disorder (AOR 1.69; 95% CI 1.14–2.50), psychosis disorder (AOR 1.63; 95% CI 1.25–2.16), depressive disorder (AOR 1.54; 95% CI 1.25–1.90) and anxiety disorder (AOR 1.41; 95% CI 1.01–1.96). Amongst the present sample, veterans were less likely than civilians to have had a recorded diagnosis of drug disorder (AOR 0.52; 95% CI 0.39–0.68).

Further analyses were conducted to explore associations between sociodemographic characteristics and recorded mental health diagnoses in veterans and civilians accessing SLaM services (Supplementary Table 2a and Supplementary Table 2b, respectively). Although these analyses did not directly compare the samples, there were some similarities in the factors associated with mental health problems across both groups. This included positive associations between psychosis disorder, ethnicity, and marital status, between stress disorder and ethnicity, between alcohol disorder and marital status, and between drug disorder and marital status. In addition, there was a negative association between alcohol disorder and ethnicity in both groups. Differences included a positive association between personality disorder and marital status, a negative association between anxiety disorder and ethnicity, and between psychosis disorder and deprivation status, amongst veterans but not civilians accessing SLaM services. Further differences included a negative association between drug disorder, ethnicity, and deprivation status, and between personality disorder and ethnicity, amongst civilians but not veterans accessing SLaM services.

## Discussion

The present study found differences in the recorded mental health diagnoses of veterans and civilians accessing secondary mental health services in South London. Depressive disorder was the most prevalent diagnosis in this sample. Whilst the most commonly recorded mental health diagnoses for veterans and civilians accessing SLaM services were alike (e.g. depressive and alcohol disorders), when compared to their civilian counterparts, veterans in South London accessing SLaM services had higher risks of many mental health problems (anxiety, depressive, personality, psychosis and stress disorders), but lower risks of drug disorder and similar risks of alcohol disorder.

The finding that veterans who were accessing SLaM services had higher risks of several mental health problems compared to civilians accessing similar services reinforces international findings that demonstrate poorer mental health outcomes amongst veterans compared to the general population [10, 12, 20, 21]. One potential explanation for this is pre-enlistment vulnerabilities, for instance veterans are more likely to have experienced childhood adversities compared to the general population [8, 9]. In addition, veterans experience specific occupational stressors related to military service, including deployment, combat exposure and the transition from military to civilian life. These experiences, in conjunction with the fact that veterans are known to wait on average 11 years after leaving service before seeking support, means that veterans are likely to have more complex mental health needs and comorbidities [22].

Alcohol disorder was the second most prevalent recorded mental health diagnosis for both veterans and civilians within the SLaM-Military-Civilian cohort. Prior research has found alcohol misuse is more common in UK Armed Forces personnel and veterans, compared to the general population [7, 10, 12]. Given the reported higher rates of alcohol misuse amongst veterans, it was surprising that this was not reflected by higher rates of accessing support for alcohol misuse amongst the present sample. The similar prevalence of alcohol disorders in civilians and veterans accessing SLaM services for mental health difficulties could potentially be related to the restricted geographical catchment area (South London) in which the cohort were accessing mental health services. For instance, SLaM NHS trust has specific addictions services and therefore patients with certain diagnoses may be referred here.

Unlike the historical acceptability of drinking in the military [23], drugs are prohibited in service [24]; this may begin to explain the lower prevalence of drug disorders in our veteran sample compared to matched civilians. A previous study reporting on the profile of a SLaM NHS Trust cohort indicated that 11.7% of their sample had a primary diagnosis of substance use disorder; a rate lower than those estimated in the current study despite including both alcohol and drug disorders [14]. There were differences in the sociodemographic profiles of the present cohort and the previous study, for instance age, gender, ethnicity, and deprivation status, which could begin to explain the different findings. A UK Government report on adult substance misuse treatment reported that more than two thirds of people in treatment were male (68% male, 32% female) [25]. As the present sample was majority male (88.4% vs. 11.6% female) compared to the previous SLaM NHS Trust cohort sample (49.5% male, 50.9% female), it is possible that the present sample included more patients with more risk factors for drug and alcohol problems.

The present study also found that veterans accessing SLaM services were at a slightly increased risk of psychosis compared to civilians after adjusting for known confounders (i.e. ethnicity and marital status). The limited evidence of psychosis in the UK Armed Forces suggest these conditions are rarer in military personnel compared to the general population; indeed, those exhibiting indicators of psychosis would not meet medical entry requirements to join the UK Armed Forces [26]. Despite this, little is known about the aetiology, onset, and trajectory of psychosis disorders in veterans, including the possibilities of psychosis as late-onset, influenced by underlying medical illness, or being substance-induced (e.g. drug-related psychosis) [27]. The overrepresentation of psychosis disorders in those with military backgrounds within the SLaM-Military-Civilian cohort could be attributed to the complexity and severity of presentations amongst those accessing secondary mental health services. Further research could examine the age of onset, age of enlistment, and type of psychosis disorder (e.g. schizophrenia or substance-induced psychosis) to better understand psychosis in this population.

Overall, the percentage of patients with psychosis disorder in both samples in the present study (8.2% civilians, 13.9% veterans) was lower than previously reported in a previous study using SLaM cohort data (21.2%) [14]. The aforementioned sociodemographic differences between the present cohort and another SLaM cohort could partly contribute to these findings. The previous study had a higher proportion of black and minority ethnic participants (45% compared to 25%). In the UK, individuals recorded under Black African and African-Caribbean ethnic categories experience a higher prevalence of psychosis compared to the White British population [28].

### Strengths and limitations

Strengths of the study include the large sample size (*N* = 2576) and the fact that the civilian and veteran samples were age- and gender-matched. CRIS draws directly from EHRs, therefore, it provides large volumes of real-world data on routine mental healthcare. This study is a step forward in understanding the specific mental health needs of veterans accessing SLaM services and how mental health problems of veteran and civilian populations accessing support for mental health conditions through SLaM may differ. In addition, the present sample was extracted from mainstream NHS secondary mental health services, whereas other research on secondary mental health care usually comes from veteran specific charities.

When interpreting the results, the study limitations need to be considered. First, the sample was taken from a single NHS Trust located in South London, meaning it is not known if the sample represents the mental health problems of civilians and military veterans accessing secondary mental health services across other UK NHS Trusts. This withstanding, the SLaM NHS Trust is one of the largest in the UK covering a geographical catchment of approximately 1.3 million people. Second, the variables explored in this study were drawn from clinical records and therefore there may be potential inconsistencies in variables across patients due to differences in disclosure and reporting by clinicians and support staff. The data analysed also relate to a specific timeframe, namely 2007 (the start of the electronic Case Register) and 2018. As a result, the analysis does not account for characteristics and mental health diagnoses outside of this timeframe. Although adverse childhood experiences appear higher in veterans compared to civilians [8, 9], the recording of childhood experiences in EHRs will likely be highly variable; for instance, clinicians may not ask or record incidents of childhood adversity, and patients may not disclose this information. This was therefore not explored in the current analyses.

Whilst all sociodemographic characteristics were backfilled to improve data completeness, there was some missing data which meant some variables could not be explored (e.g. employment status). Some missing data might be due to certain details not being routinely collected in EHRs (e.g. employment status), and/or it might be the result of limitations of the CRIS system which only started in 2007 despite EHRs starting in 2003. Therefore, potentially not all data had been entered into the system. It is not expected that this would be different for veterans and civilians, instead it is anticipated that they would be affected to the same degree. Additionally, it was not possible to separate certain disorders of interest or to ascertain whether the diagnoses represented current or lifetime diagnoses; only that they were the diagnoses on record within the reporting period spanning 2007–2018.

Furthermore, due to the descriptive and exploratory nature of this paper, samples used in the present study were only matched on age and gender. These factors were chosen because they are salient in the military context because they are associated with being part of the military and transitioning to being a veteran, with the prevalence of mental health problems, and with engagement with mental health services. Other approaches, including matching on further factors, could have been implemented. However, some of these other factors (for example employment status and benefit status) had a high degree of missing data for this study’s sample.

Some of the reported associations in Supplementary Tables 2a and 2b should be interpreted with caution due to the analyses being statistically underpowered (*n* < 10). Lastly, the study was limited in assessing the influence of military characteristics, such as service branch (which had > 50% missing data), length of service, deployment exposures or length of time between leaving the military and accessing secondary mental health care services.

### Implications

This study found that military veterans accessing secondary mental health services had higher risks for most mental health problems compared to matched civilians. Although these findings may not be generalisable to the wider UK population, it appeared veterans represent a subgroup of the population accessing SLaM services with different mental health needs than civilians. The finding that veterans accessing support in SLaM NHS Trust are more at risk of many mental health problems highlights the importance of improving the reporting of military service history both for research purposes and to support clinicians in providing appropriate treatment to veterans. Clinicians should recognise that veterans have complex mental health needs which differ to those of civilians. Therefore, asking about veteran status and conducting a thorough assessment, are important to tailor interventions to meet veterans’ unique needs.

## Conclusions

To conclude, this study found differences in the recorded mental health diagnoses of military veterans and civilians accessing secondary mental health services in South London. In this Trust, veterans had higher rates of a range of mental health problems, except from alcohol and drug disorder, when compared to matched civilians. The finding that veterans may have different mental health needs compared to civilians adds to the body of literature and has clinical implications. These findings should be corroborated using data from other NHS Trusts, and researchers could consider the possibility of creating larger population-based studies of UK veterans’ mental health using EHRs.

## Supplementary Information

Below is the link to the electronic supplementary material.

Supplementary file1 (DOCX 13 KB)

Supplementary file2 (DOCX 19 KB)

Supplementary file3 (DOCX 19 KB)

## Data Availability

The datasets used in the current study are based on pseudonymised patient data which are not publicly available. Access to this data requires a formal application to the Patient Data Oversight Committee of the National Institute of Health Research Biomedical Research Centre. On request, and after suitable arrangements are put in place, the data and modelling employed in this study can be viewed within the secure system firewall.
