# Supplementary material for: Military veterans and civilians’ mental health diagnoses: an analysis of secondary mental health services
Source: Soc Psychiatry Psychiatr Epidemiol. 2022 Dec 22;58(7):1029–37. doi: 10.1007/s00127-022-02411-x (PMC10261174; doi:10.1007/s00127-022-02411-x)
Supplement: Supplementary file 1 — Supplementary file1 (DOCX 13 KB) [file 127_2022_2411_MOESM1_ESM.docx]

**Supplementary Table 1.** Diagnostic Grouping of ICD-10 Coding Used in this Study

| **Diagnosis Group** | **ICD-10 Coding** |
| --- | --- |
| Alcohol disorder | **F10:** Mental and behavioural disorders due to use of alcohol |
| Anxiety disorder | **F40:** Phobic anxiety disorders  **F41:** Other anxiety disorders  **F42:** Obsessive-compulsive disorder  **F48:** Other neurotic disorders |
| Depressive disorder | **F30­-F39:** Mood [affective] disorders |
| Drug disorder | **F11:** Mental and behavioural disorders due to use of opioids  **F12:** Mental and behavioural disorders due to use of cannabinoids  **F13:** Mental and behavioural disorders due to use of sedatives or hypnotics  **F14:** Mental and behavioural disorders due to use of cocaine  **F15:** Mental and behavioural disorders due to use of other stimulants, including caffeine  **F16:** Mental and behavioural disorders due to use of hallucinogens  **F17:** Mental and behavioural disorders due to use of tobacco  **F18:** Mental and behavioural disorders due to use of volatile solvents  **F19:** Mental and behavioural disorders due to multiple drug use and use of other psychoactive substances |
| Personality disorder | **F60:** Specific personality disorders  **F61:** Mixed and other personality disorders |
| Psychosis disorder | **F20-F29:** Schizophrenia, schizotypal and delusional disorders |
| Stress disorder | **F43:** Reaction to severe stress, and adjustment disorders |
| Other disorders | All other ‘F’ codes not denoted above |
| Diagnosis not specified | All other ICD-10 codes not mentioned above (excluding ‘F’ and ‘Z’ codes) |
