# Supplementary material for: Military veterans and civilians’ mental health diagnoses: an analysis of secondary mental health services
Source: Soc Psychiatry Psychiatr Epidemiol. 2022 Dec 22;58(7):1029–37. doi: 10.1007/s00127-022-02411-x (PMC10261174; doi:10.1007/s00127-022-02411-x)
Supplement: Supplementary file 3 — Supplementary file3 (DOCX 19 KB) [file 127_2022_2411_MOESM3_ESM.docx]

**Supplementary Table 2a. Associations between Socio-Demographic Characteristics and Recorded Mental Health Diagnoses in Civilians (*n*=1,288)**

|  | **Alcohol disorder**  **(F10)** | | **Anxiety disorder**  **(F40, F41)** | | **Depressive disorder**  **(F32, F33)** | | **Drug disorder**  **(F11-F19)** | | **Personality disorder**  **(F60-F69)** | | **Psychosis disorder**  **(F20-F29)** | | **Stress disorder**  **(F43)** | |
| --- | --- | --- | --- | --- | --- | --- | --- | --- | --- | --- | --- | --- | --- | --- |
|  | **n(%)** | **AOR**  **(95% CI; *p* value)^1^** | **n(%)** | **AOR**  **(95% CI; *p* value)^1^** | **n(%)** | **AOR**  **(95% CI; *p* value)^1^** | **n(%)** | **AOR**  **(95% CI; *p* value)^1^** | **n(%)** | **AOR**  **(95% CI; *p* value)^1^** | **n(%)** | **AOR**  **(95% CI; *p* value)^1^** | **n(%)** | **AOR**  **(95% CI; *p* value)^1^** |
| Ethnicity  White  Non-white | 147 (19.2)  25 (9.5) | 1  0.49 (0.31 to 0.77; 0.002)* | 51 (6.7)  13 (5.0) | 1  0.70 (0.36 to 1.33; 0.266) | 143 (18.7)  44 (16.8) | 1  0.89 (0.61 to 1.30; 0.546) | 128 (16.8)  33 (12.6) | 1  0.65 (0.42 to 0.99; 0.047)* | 37 (4.8)  5 (1.9) | 1  0.33 (0.13 to 0.86; 0.023)* | 47 (6.2)  56 (21.4) | 1  4.98 (3.16 to 7.84; <.001)* | 34 (4.5)  19 (7.3) | 1  1.82 (1.00 to 3.31; 0.050)* |
| Marital status  Married/Relationship  Single/Separated/  Divorced/Widowed | 31 (13.7)  137 (19.0) | 1  1.70 (1.11 to 2.62; 0.016)* | 18 (7.9)  44 (6.1) | 1  0.75 (0.42 to 1.33; 0.324) | 49 (21.6)  137 (19.0) | 1  0.85 (0.58 to 1.23; 0.377) | 21 (9.3)  134 (18.6) | 1  2.13 (1.31 to 3.49; 0.003)* | 4 (1.8)  37 (5.1) | 1  2.46 (0.86 to 7.06; 0.093) | 8 (3.5)  87 (12.1) | 1  4.00 (1.90 to 8.43; <.001)* | 11 (4.9)  39 (5.4) | 1  1.06 (0.53 to 2.13; 0.871) |
| Living arrangements  Alone  With others^2^ | 82 (26.0)  70 (16.6) | 1  0.70 (0.46 to 1.08; 0.109) | 18 (5.7)  28 (6.7) | 1  1.04 (0.49 to 2.18; 0.922) | 60 (19.0)  84 (20.0) | 1  0.95 (0.68 to 1.32; 0.754) | 74 (23.4)  65 (15.4) | 1  0.69 (0.45 to 1.06; 0.093) | 16 (5.1)  16 (3.8) | 1  0.80 (0.36 to 1.77; 0.582) | 35 (11.1)  37 (8.8) | 1  1.40 (0.82 to 2.41; 0.217) | 16 (5.1)  22 (5.2) | 1  0.94 (0.42 to 2.10; 0.880) |
| Deprivation status^3^  Most deprived (high)  Least deprived (low to middle) | 93 (16.4)  80 (15.2) | 1  0.96 (0.68 to 1.35; 0.793) | 29 (5.1)  39 (7.4) | 1  1.56 (0.93 to 2.64; 0.095) | 103 (18.2)  88 (16.7) | 1  0.95 (0.68 to 1.32; 0.754) | 95 (16.8)  61 (11.6) | 1  0.66 (0.46 to 0.95; 0.025)* | 23 (4.1)  17 (3.2) | 1  0.84 (0.44 to 1.61; 0.593) | 63 (11.1)  40 (7.6) | 1  0.76 (0.49 to 1.19; 0.232) | 34 (6.0)  21 (4.0) | 1  0.77 (0.43 to 1.39; 0.390) |

**Notes.** AOR: adjusted odds ratios; CI: confidence intervals

^1^Regression models adjusted for age (in bands of 10 years), and marital status.

^2^This includes staying with foster parents and friends.

^3^The Index of Multiple Deprivation is the official measure of relative deprivation for small areas (or neighbourhoods) in England. The Index of Multiple Deprivation ranks every small area in England from 1 (most deprived area) to 10 (least deprived area) based on a range of factors.

^*^*p* < 0.05
